# Job Seeking While Caregiving: Is There a Hiring Penalty for Family Caregivers?

**DOI:** 10.1093/geroni/igaf122.592

**Published:** 2025-12-31

**Authors:** Samantha Brady

**Affiliations:** Massachusetts Institute of Technology, Cambridge, Massachusetts, United States

## Abstract

Whether by choice or necessity, the majority of unpaid family caregivers to adults in the U.S. are employed. Prior research highlights the importance of work for caregivers’ financial stability, emotional well-being, and physical health. However, less is known about how caregiving responsibilities for an adult affect job seekers, particularly in hiring decisions. While hiring bias against parents is well-documented, the impact of adult caregiving on employment opportunities remains under-explored. Using data from an original hiring experiment of hiring managers across the US, we examine how family caregivers are perceived in the hiring process and whether they face penalties due to their caregiving roles. Results show that unpaid family caregivers are 4.4 percentage points less likely to be hired than equally qualified applicants without caregiving responsibilities and 2.7 percentage points less likely to be hired than applicants with dependent children, controlling for age, gender, education, and skill level. Further analysis reveals that while both parents and caregivers to adults are seen as less available and reliable than non-caregivers, caregivers to adults are also perceived as less committed, contributing to their lower hiring rates. While existing research documents workplace challenges for employed caregivers, our findings underscore an earlier and often overlooked barrier: the hiring process itself. These hiring penalties may limit career mobility and threaten the financial security of millions of unpaid caregivers, raising critical implications for employment policies and workplace equity.

